# Better survival in female *SOD1*-mutant patients with ALS: a study of *SOD1*-related natural history

**DOI:** 10.1186/s40035-018-0142-8

**Published:** 2019-01-08

**Authors:** Lu Tang, Yan Ma, Xiao-lu Liu, Lu Chen, Dong-sheng Fan

**Affiliations:** Department of Neurology, Peking University Third Hospital, 49 North Garden Road, Haidian District, Beijing, 100191 People’s Republic of China

**Keywords:** Amyotrophic lateral sclerosis, Natural history studies, *SOD1* mutations, Gender difference

## Abstract

**Background:**

*SOD1* mutations are the most common cause of amyotrophic lateral sclerosis (ALS) in non-Caucasian patients. Detailed natural history profiles of *SOD1*-mutant patients will be beneficial for the strategy and interpretation of future *SOD*1-targeted clinical practice.

**Methods:**

Mutational distribution, age at onset (AAO), site of onset, diagnostic delay, disease progression (rate of ALSFRS-R decrease, ΔFS) and survival were analysed. Further comparisons between heredity of disease, gender, and mutations were performed.

**Results:**

Sixty-six cases with 43 *SOD1* mutations were included and analysed, with p.His47Arg as the leading mutation and seven novel variants identified. The mean (SD) AAO was 43.92 years (9.24) for all subjects, with a significant difference between patients carrying mutations in exon 2 (*n* = 24,46.83, 8.31) and exon 4 (*n* = 18, 37.75, 7.67) (*p* = 0.002). The median (IQR) diagnostic delay from symptom onset was 14.50 (6.00–36.50) months for all *SOD1*-mutant patients, 9.50 (4.75–24.25) months for males and 24.00 (9.50–47.50) months for females, revealing a gender difference (*p* = 0.009). Similar advantages in median (IQR) ΔFS [male: female, 0.55 (0.24–0.94) vs 0.19 (0.06–0.90), *p* = 0.041] and mean (95% CI) survival [57.4 (38.90–75.90) months vs 125.6 (99.80–151.50) months, *p* = 0.006] were also observed in females, both of which existed in sporadic ALS only when stratified by familiar or sporadic ALS.

**Conclusions:**

The results highlight a distinct mutational distribution and natural history spectrum in ALS patients carrying *SOD1* mutations in China. A prominent mild disease progression was observed in female patients, which had rarely been reported in the previous literature. This finding, together with the detailed analysis of natural history among each mutation, can have important implications for future genetic counselling and *SOD1*-targeted clinical trials.

## Background

Amyotrophic lateral sclerosis (ALS) is a fatal neurodegenerative disorder involving the motor system with a progressive, fatal course within 3–5 years after onset. Most cases of ALS are sporadic (sALS), while approximately 5–10% cases are familial ALS (fALS). ALS-causing mutations have been so far identified in more than 25 genes, including Cu/Zn superoxide dismutase 1 (*SOD1*) [1], TAR DNA-binding protein-43 (*TARDBP*) [2, 3], fused in sarcoma (*FUS*) [4, 5], optineurin (*OPTN*) [6], chromosome 9 open reading frame 72 (*C9orf72*) [7, 8], and sequestosome 1 (*SQSTM1*) [9]. Although *C9orf72* is the predominant mutant gene in the Caucasian ALS population, it is rather rare in non-Caucasian patients with ALS [10, 11]. Mutations in *SOD1* are one of the most common and important causes of ALS, accounting for 23% of fALS and ~ 7% of apparently sALS worldwide [12]. More than 185 mutations in *SOD1* have been reported to date [13]. Multiple efforts have focused on targeted therapeutic approaches for *SOD1*-related ALS (ClinicalTrials.gov: NCT01041222: SOD1Rx [14]; NCT00706147: Arimoclomol [15]; NCT01083667: Pyrimethamine [16]).

In the present study, we described the genetic and natural history profiles of ALS patients with *SOD1* mutations obtainted from a national referral hospital site in China. The effort to clinically stratify mutations in *SOD1* according to the patients’ natural histories should be beneficial for future genetic counselling and the selection of genetically and clinically homogeneous patients for *SOD1*-targeted clinical trials for ALS.

## Methods

### Subjects

Patients were recruited from the national referral Amyotrophic Lateral Sclerosis Clinic at the Department of Neurology, Peking University Third Hospital (PUTH), Beijing from 2007 to 2013. The patients were examined and diagnosed by board-certified neurologists as having definite, probable, or possible ALS according to Airlie House diagnostic criteria [17]. All patients included provided written informed consent to participate in the clinical and genetic studies, which were approved by the institutional ethics committee of PUTH, during their first visit to the hospital. Only patients with genetically confirmed *SOD1* mutations/variants were included in further clinical analysis.

### Procedure

#### Clinical data collection and analysis

Demographic and clinical patient data, including gender, age at onset (AAO), site at onset, diagnostic delay, and ALS Functional Rating Scale - Revised (ALSFRS-R) score at diagnosis, were collected during the first visit to the hospital,. Outcome/endpoint event data were updated during telephone follow-ups every three months. Diagnostic delay from onset was identified as the interval from symptom onset to diagnosis. Disease progression was defined as the rate of decrease in the ALSFRS-R score at enrolment (ΔFS), which was calculated as follows: ΔFS = (48-ALSFRS-R score at diagnosis) / diagnostic delay (months) [18]. Cut-off ΔFS values of 0.5 and 1.0 were applied to divide the patients into three subgroups: slow progression (< 0.5), intermediate progression (0.5–1.0), and fast progression (> 1.0) [19, 20]. Survival time was defined as the interval from symptom onset to endpoint events from any cause or the last follow-up, where death and tracheotomy were defined as endpoint events. The censoring date for survival data was May 31, 2016. Patients lost to follow-up were censored at the last known living data point. Additional clinical features, such as cognitive status and presence of sensory symptoms and signs, were not analysed in this study.

#### Genetic analysis

Only probands of families and apparently sporadic patients were included in the genetic detection analysis. Sanger sequencing was performed for all coding exons and flanking 50 bps of SOD1 (NM_0000454.4) and sequential FUS [NM_004960.3] and TARDBP [NM_007375.3] for patients with variants in *SOD1*. Moreover, expansions in *C9orf72* were detected using repeat-primed PCR as previously described [10]. Variants were reported using the HGVS-Sequence Variant Nomenclature. Mutations were researched in the SNP database (dbSNP, http://www.ncbi.nlm.nih.gov/projects/SNP) [21], Exome Aggregation Consortium (ExAC) (http://exac.broadinstitute.org) [22], 1000 Genomes Project (http://www.internationalgenome.org) [23], ALSod (http://alsod.iop.kcl.ac.uk/) [13], and HGMD (http://www.hgmd.cf.ac.uk/ac/index.php) [24] as references. The detrimental role of novel mutations was predicted with the Mutation Taster (http://www.mutationtaster.org/index.html) [25], PolyPhen-2 (http://genetics.bwh.harvard.edu/pph2/) [26], and PROVEAN (http://provean.jcvi.org/index.php) [27] bioinformatics prediction tools. The novel variants identified in the present study were evaluated according to the American College of Medical Genetics (ACMG) Standards and Guidelines [28].

### Statistical analysis

The mean (SD) AAO, median (IQR) diagnostic delay, mean (IQR) ΔFS, and mean (SD) disease duration were straightforwardly calculated [29] for each mutation. Descriptive statistics were provided for the bulbar site of onset, AAO, diagnostic delay, ΔFS and survival time by total patients, heredity of disease (familial or sporadic ALS), and gender. Moreover, Student’s t-test or the Mann-Whitney test was applied to compare continuous data, while a standard chi-square test or Fisher’s exact test was used to analyse dichotomous variables, such as gender and site of onset. Survival time was determined using Kaplan-Meier analysis, and differences were determined by log-rank testing. A two-tailed *p* < 0.05 was considered statistically significant. All analyses were performed using GraphPad Prism 5.0 (GraphPad Software, CA, USA).

## Results

### Overview of demographic and genetic features

A total of 923 patients with sALS (male: female, 600:323, 1.86) and 159 with fALS (male: female, 89:70, 1.27) were successfully screened for the *SOD1* gene. Among them, 66 subjects were genetically identified as having *SOD1* mutations/variants, 47 (47/159, 30%) of whom were fALS patients and 19 (19/923, 2%) of whom were apparently sALS patients; none of these patients were positive for *FUS*, *TARDBP*, or *C9orf72* mutations. Except for one case with missing data, the gender ratio (M: F) was 1.2:1, showing a slight male dominance. Five patients among 62 available patients (5/62, 8%), including 3 familial and 2 sporadic cases, exhibited bulbar sites of onset.

A total of 39 missense, 1 nonsense, and 3 deletion/insertion mutations/variants were found spanning all five exons, only one of which was in exon 3. The most frequent *SOD1* mutation was p.His47Arg, traditionally named H46R, which was found in 6 fALS patients and 2 sALS patients (8/66, 12%), followed by p.Gly42Asp, p.Gly42Ser, p.His44Arg, p.Leu107Phe, and p.Gly142Ala in 3 patients each (3/66, 4.5%). Seven novel variants, including p.Phe21Val, p.Trp33Gly, p.Arg80Ser, p.Gly86Cys, p.Ala90Phe, p.Val95Gly, and p.Glu133Ter, were identified as variants of unknown significance (VUS) or likely pathogenic variants in the present study (Table 1). Some of the *SOD1* mutations identified in fALS patients were previously reported [30].

**Table 1:** Novel variants of the SOD1 gene found in the present study but absent in reference databases

Exon	DNA changes	Amino acid changes	Case count	Hereditary	ACMG score	Any report in the same codon from reference database	Found in ExAC or 1000G	Mutation prediction		
								MutationTaster	PolyPhen-2	PROVEAN
Exon 1	c.61 T > G	p.Phe21Val	1	fALS	VUS	p.Phe21Cys	neither	disease causing	probably damaging	damaging
Exon 2	c.97 T > G	p.Trp33Gly	1	sALS	VUS	none	neither	polymorphism	benign	tolerated
Exon 4	c.240G > T	p.Arg80Ser	1	fALS	VUS	none	neither	disease causing	probably damaging	damaging
Exon 4	c.256G > T	p.Gly86Cys	1	fALS	VUS	p.Gly86Arg/p.Gly86Ser	neither	disease causing	probably damaging	damaging
Exon 4	c.268_269delinsTT	p.Ala90Phe	1	fALS	VUS	p.Ala90Thr/p.Ala90Val	neither	disease causing	probably damaging	damaging
Exon 4	c.284 T > G	p.Val95Gly	1	sALS	Likely Pathogenic	p.Val95Ala	neither	disease causing	probably damaging	damaging
Exon 5	c.397G > T	p.Glu133Ter	1	sALS	Likely Pathogenic	p.Glu133Lys/ p.Glu133insTT/p.Glu133delGAA	neither	disease causing	NA	NA

*ACMG*, American College of Medical Genetics and Genomics. *ExAC*, Exome Aggregation Consortium. *VUS*, variant of unknown significance

We found that the mutation p.His47Arg exhibited a relatively consistent and mild phenotype, expect for in a male patient who was 69 years old at onset and died within 14 months after onset. The other seven patients presented a mean (SD) AAO of 48.14 (7.47) years, a median (IQR) diagnostic delay of 62 (45–84) months, and a disease course between 65 and 155 months, with no endpoint events.

### Age at disease onset

**Fig. 1:**
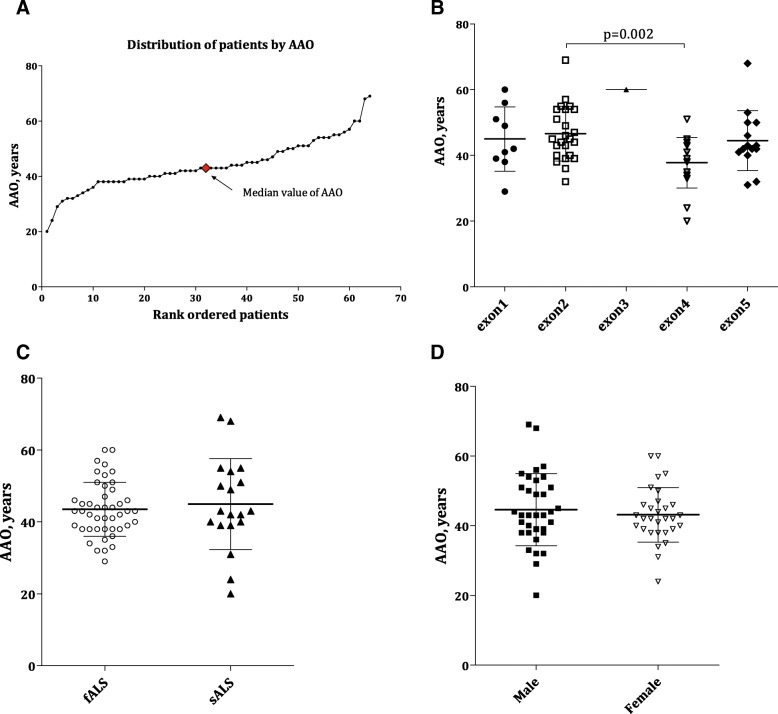
Age at onset (AAO) of patients with SOD1 mutations. **a** Plot of rank ordered *SOD1*-mutant patients showing the median AAO of 43 years. **b** Plot comparing the AAO among patients carrying mutations in different exons of the *SOD1* gene. Patients (*n* = 24) harbouring mutations in exon 2 were older than those (*n* = 18) harbouring mutations in exon 4 (46.63 years vs 37.75, *p* = 0.002). **c** Plots comparing fALS patients with sALS patients and (**d**) plots comparing male patients with female patients; neither comparison identified a significant difference in AAO (*p* = 0.64 and 0.53, respectively)

Sixty-four patients had available data for AAO analysis. The mean (SD) AAO for all patients was 43.92 (9.24) years, and the median (IQR) AAO was 43.00 (38.25–50.00) years. A significant difference in the mean (SD) AAO was observed between patients carrying mutations in exon 2 (*n* = 24,46.63, 8.31) and those with mutations in exon 4 (*n* = 18, 37.75, 7.67) (*p* = 0.002) (Fig. 1), whereas there was no difference in the mean (SD) AAO between fALS and sALS patients [43.49 (7.50) vs 44.95 (12.63), *p* = 0.64] or between male and female patients [44.62 (10.38) vs 43.13 (7.84), *p* = 0.53] (Table 3, Fig. 1).

The AAO varied among different mutations, with p.Asn140Lys and p.Ser106Leu being associated with the oldest and youngest AAOs (patients in their sixties and twenties, respectively), albeit with only one case for each mutation (Table 2). Ten mutations/variants presented a relatively older AAO (more than the third quartile of 50 years, highlighted in green in Table 2), and eleven showed relatively younger AAO (less than the first quartile of 38.25 years, highlighted in yellow in Table 2).

**Table 2:**
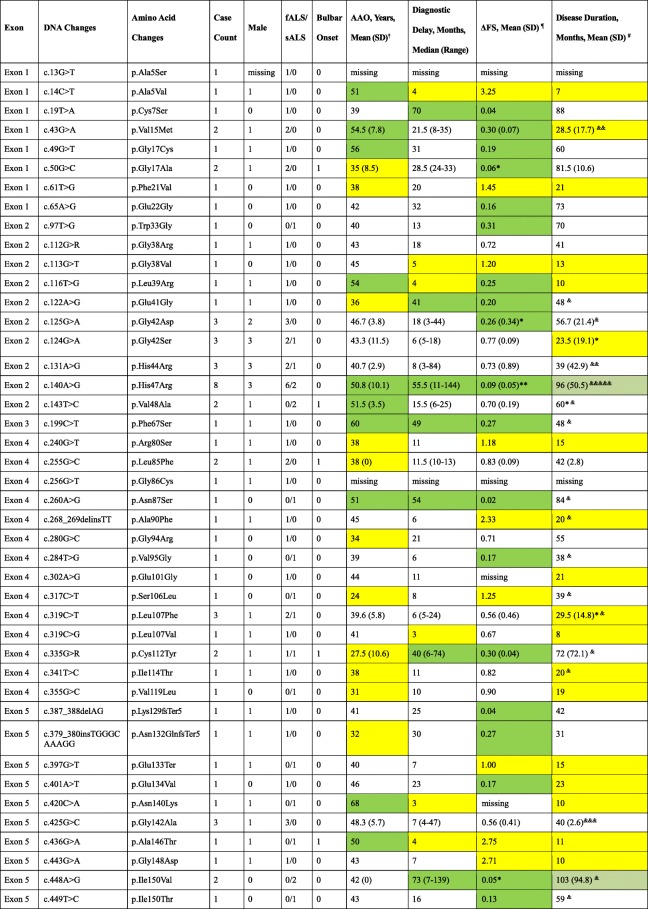
Clinical features according to the mutation/variant type in SOD1-mutant patients

*There was one case with missing data. ** There were two cases with missing data

& There was one surviving case. && There were two surviving cases. &&& There were three surviving cases. &&&&& There were five surviving cases

† The interquartile ranges (IQRs) of age at onset and diagnostic delay were calculated and determined to be 38.25–50 years and 6–36.50 months, respectively. Values greater than the third quartile (75%) are highlighted in green, whereas values less than the first quartile (25%) are highlighted in yellow

**¶** The disease progression was calculated as ΔFS = (48 - ALS-FRSR at enrolment) / (diagnostic delay in months). Based on references [21] and [22], values less than 0.50 were defined as slow progression and highlighted in green, whereas values greater than 1.00 were defined as fast progression and highlighted in yellow

# For disease duration, there were 61 available cases, and 60% were censored (lost to follow-up or still surviving). Therefore, 25% of the patients survived less than 31 months, and 50% of the patients survived less than 89 months. Values greater than the second quartile (89 months) are highlighted in light green, whereas values less than the first quartile (31 months) are highlighted in yellow

### Diagnostic delay from onset

Diagnostic delay information was available for 64 patients. The median (IQR) diagnostic delay from onset was 14.50 (6.00–36.50) months for all subjects, 20.00 (6.50–39.00) months for fALS patients, and 8.00 (6.00–25.00) months for sALS patients. There was a significant difference in the median diagnostic delay between males (9.50, 4.75–24.25) and females (24.00, 9.50–47.50) (*p* = 0.009) (Table 3). In both fALS and sALS patients, the difference in diagnostic delay between genders was significant (male vs female, median in fALS: 13.00 vs 32.50, *p* = 0.042; male vs female, median in sALS: 6.00 vs 14.50, *p* = 0.027).

**Table 3:** Demographic and clinical features of familial and sporadic ALS patients with *SOD1* mutations

	Case count, n (%)	Bulbar site at onset, n (%)	AAO, years, mean (SD)	Delay, months, median (IQR)	Disease progression, ΔFS, median (IQR)	Survival time, months, mean (95% Cl)^&^
Total	66	5(8.1%)	43.92(9.24)	14.50(6.00–36.50)	0.33 (0.15–0.90)	97.08 (75.61–118.54)
Missing	–	4	2	2	9	5
fALS	47(71.2%)	3(7.0%)	43.49(7.50)	20.00(6.50–39.00)	0.27(0.15–0.87)	87.46(61.74–113.18)
sALS	19(28.8%)	2(10.5%)	44.95(12.63)	8.00(6.00–25.00)	0.58(0.14–0.98)	121.49(86.24–156.74)
*p* value	–	0.638	0.64	0.26	0.59	0.38
Male	35(53.8%)	4(11.8%)	44.62(10.38)	9.50(4.75–24.25)	0.55(0.24–0.94)	57.40(38.89–75.91)
Female	30(46.2%)	1(3.6%)	43.13(7.84)	24.00(9.50–47.50)	0.19(0.06–0.90)	125.64(99.83–151.45)
p value	-^#^	0.366	0.525	0.009*	0.041*	0.006*

AAO, age at onset. Delay, diagnostic delay from onset

& Due to more than 50% of subjects being censored (lost to follow-up or still surviving) in the sALS and female groups, the mean (95% CI) survival time was calculated

# There was one case without gender information

*In bold with statistical significance at p < 0.05

Diagnostic delay varied greatly across different mutations. Six mutations, namely, p.Ala5Val, p.Gly38Val, p.Leu39Arg, p.Leu107Val, p.Asn140Lys, and p.Ala146Thr, showed a diagnostic delay less than the first quartile (25%, < 6 months). Another seven mutations, namely, p.Cys7Ser, p.Glu41Gly, p.His47Arg, p.Phe67Ser, p.Asn87Ser, p.Cys112Tyr, and p.Ile150Val, showed a diagnostic delay more than the third quartile (75%, > 36.5 months) (highlighted in yellow and green in Table 2, respectively).

### Disease progression

Because ALSFRS-R scores at enrolment were available for 57 patients, the rate of disease progression was analysed in these patients. The median (IQR) ΔFS was 0.33 (0.15–0.90)/month. There was no significant difference in ΔFS between fALS and sALS patients [0.27 (0.15–0.87) vs 0.58 (0.14–0.98), *p* = 0.59). However, female patients manifested a slower progression, with a median (IQR) ΔFS of 0.19 (0.06–0.90), than males (0.55, 0.24–0.94) (*p* = 0.041) (Table 3). Further comparison showed that female patients presented a significantly slower progression of sALS (male vs female: 0.83 vs 0.17, *p* = 0.031) but not of fALS (0.33 vs 0.23, *p* = 0.29).

When the categories of disease progression (slow, intermediate, and fast) were applied with ΔFS values of 0.5 and 1.0, 30 patients (30/57, 53%) carrying 22 different mutations were identified with slow progression (highlighted in green in Table 2), 18 patients (18/57, 32%) carrying 12 mutations were identified with intermediate progression, and 9 patients (9/57, 16%) carrying 9 mutations deteriorated more rapidly (highlighted in yellow in Table 2).

### Disease duration and survival

**Fig. 2:**
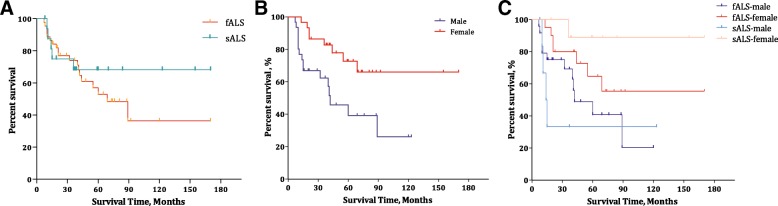
Survival analysis of fALS and sALS patients carrying *SOD1* mutations and comparison between genders. **a** Plot of survival probabilities for fALS vs sALS patients with *SOD1* mutations; no significant differences were observed (87.5 vs 121.5, *p* = 0.382). **b** Plot of survival probabilities between genders, indicating that females had a longer survival time than males (125.64 vs 57.40, *p* = 0.006). Another plot (**c**) was made to compare the survival function between genders in fALS and sALS patients; a significant difference of *p* = 0.035 was observed

Course of disease data were available for 61 subjects, including 12 subjects censored for lost to follow-up and 26 subjects still surviving at the censoring date. Based on statistical analyses, the median survival time was 89.0 months, the mean survival time was 97.1 (95% Cl 75.6–118.5) months, and the 5-year survival rate was 55% for all subjects. For insufficient endpoint events, the mean (95% CI) survival time was calculated between groups, which was 87.5 (61.7–113.2) months and 121.5 (86.2–156.7) months for patients with fALS and sALS, respectively, with no difference between these patients (*p* = 0.38). Female patients showed a significantly longer survival time (125.64 months, 99.8–151.5 months) than male patients (57.40 months, 38.89–75.91 months) (*p* = 0.006) (Table 3). Therefore, female patients had a better 5-year survival rate (72%) than male patients (37%). When genders were compared among the different heredity groups, a significant difference in survival time was detected in sALS patients [male vs female, 49.33 months (7.63–91.04) vs 155.11 months (127.60–182.62), *p* = 0.009)] but not in fALS patients [57.73 (38.45–77.01) vs 111.92 (78.48–145.35), *p* = 0.10] (Fig. 2).

Among patients with different mutations, those with p.Ala5Val and p.Ile150Val had the shortest and longest disease durations, respectively; notably, the latter group was still alive at the censoring date. Because 60% of the cases were censored, we were able to only calculate only the first and second quartiles of disease duration as 31.0 months (mutations highlighted in yellow, Table 2) and 89.0 (mutations highlighted in light green, Table 2), respectively.

## Discussion

We identified 66 patients harbouring 43 confirmed *SOD1* mutations among 923 sALS and 159 fALS patients. Because of the number of patients available, the present study represents the largest effort to study *SOD1* mutations to date in a non-Caucasian ALS population, including 47 fALS patients and 19 sALS patients with *SOD1* mutations. The extensive survey and analysis of ALS-related *SOD1* mutations and the patients’ natural histories provide data that could serve in the design and interpretation of future clinical trials targeted at patients with *SOD1* mutations.

In Chinese *SOD1* mutant patients with ALS, p.His47Argwas most frequently identified (8/66, 12%); this mutation is found mainly in Asian ALS patients (more than 15 pedigrees have been reported, only 3 of which are of Caucasian descents) [31]. P.His47Arg was also the leading mutation in Japanese *SOD1*-mutant patients [32]. In our study, almost all patients with p.His47Arg presented with the characteristic phenotype of exclusive spinal muscle initiations but mild disease course, i.e., an older AAO, extended diagnostic delay, slower progression, and longer survival time (Table 2), with the exception of one patient with a survival time of only 14 months. Conversely, the most globally predominant *SOD1* gene mutation, D90A [32], was absent in our study, and only one case with A4V (p.Ala5Val, 1/66) and one case with I133T (p.Ile114Thr, 1/66) were found; these two mutations account for ~ 50% and ~ 65% of *SOD1* mutations in the United States [29] and Canada [33], respectively. This result is not uncommon since the frequency of specific mutations can vary among different countries or even among different regions of the same country. For example, the A4V mutation has also been rarely observed in Europe. Another case in point is the mutation cluster of L48F-*SOD1*, which has been described in central Italy but is quite rare in other Italian regions [34].

Of note is the gender difference in patients harbouring *SOD1* mutations. Male predominance was present but mild in *SOD1*-mutant patients (M: F of 1.2 overall, 1.3 in fALS, and 0.9 in sALS); these ratios are lower than those in other reports from China (5/3 = 1.6) [35], Taiwan (7/5 = 1.4) [36], and Iran (4/3 = 1.3) [37] despite the small sample sizes in those studies. These ratios are also lower than those in both the present cohort (689/393 = 1.75) and in the ALS population in China (1.47–1.7) [35, 38]; however, they are comparable to that of *SOD1*-mutant patients found in the United States (1.3, 175 fALS included) [29].

Furthermore, significant beneficial effects of female gender on diagnostic delay, disease progression and survival time were observed, and we postulate two explanations for this result. 1) The mutations with poorer prognosis presented more often in male patients. For example, 17 patients had a diagnostic delay of less than 6 months (the first quartile), 13 of which were males; similarly, 13 patients had an ΔFS of more than 0.90 (the third quartile), 8 of which were males. Finally, 23 patients had a survival time of less than 31 months (the first quartile), 16 of which were males. 2) For the identical mutation, the male patients presented a poorer prognosis than females. In total, 8 patients carried the p.H47R mutation. Among these patients, the 3 male patients presented a mean diagnostic delay of 54.7 months, an ΔFS of 0.12, and a survival time of 70 months, while these values in the other 5 female patients were 67.4 months, 0.08, and 120.5 months, respectively. The difference in the diagnostic delay between genders existed in both fALS (*p* = 0.042) and sALS (*p* = 0.027), while gender differences in disease progression (*p* = 0.031) and survival time (*p* = 0.009) were present in only sALS. The finding of a longer survival time in female patients is consistent with what we previously found in the overall sALS population (female: male, 87 months vs 63 months, *p* = 0.008) [38]. To our knowledge, such a gender difference in *SOD1*-related ALS patients’ natural histories has not been previously described. The rarity of this finding may be due to having an insufficient number of ALS cases with *SOD1* mutations or a lack of detailed follow-up data. It remains unclear why female patients present a more remarkable survival advantage in the sporadic population. A possible explanation for this discrepancy is that fALS is inherited as a mendelian disease (monogenic), while sALS is likely an oligogenic disease [39]. We speculate that in fALS cases, the impact of a causative gene is predominant; while in sALS cases, the effect of each variant in certain gene is minor, and the modifying effect of gender could therefore be manifested. Since extended diagnostic delay has been associated with longer survival in ALS patients in several studies [38, 40], it is reasonable that females with a longer diagnostic delay had better survival in the present study. The effect of gender on survival time has been mentioned multiple times with contrasting findings in ALS populations, with some studies suggesting worse survival for women [41, 42] and others suggesting better survival [43, 44]. One possible explanation for gender differences could be the role of gonadal hormones since these hormones, particularly oestrogen, have been proven to be neuroprotective. A very recent report [45] demonstrated a negative association between ALS and hormonal contraception use in women, reporting a dose-response effect. Another potential reason for the gender difference is that smoking is a risk factor for ALS; patients who smoke may have a shorter survival time [46, 47], and there is a higher percentage of male smokers in the Chinese ALS population than female smokers (male: 44.7% vs female: 1.7%) [38]. Another supporting and interesting interpretation is from a meta review [48] of a *SOD1* G93A mouse model of ALS. That report was suggestive of gender- and genetic background-related effects on disease course, i.e., female-related neuroprotective effects on lifespan and disease duration were observed for B6SJL mice but not for mice on a C57BL/6 background, implying that the inherent genetic differences observed between backgrounds had some interactive effect on the presentation of the female hormone-related protective effect.

The mean AAO of the *SOD1*-mutant patients in this study was 43.92 (95% Cl 41.61–46.23) years, which was younger than that of 45.5 years reported in a southwest China [35] study that identified only 8 patients carrying mutant *SOD1* genes among 499 ALS patients. This mean AAO is also younger than that of the overall Chinese ALS population (49.7, 95% Cl 49.2–50.3) [38] and younger than that of *SOD1*-mutant patients reported in Canada (48.9) [49] and the United States (46.9–49.7) [29, 50]. As described in a previous study, *SOD1*-mutant patients in a specific population were also younger than the overall ALS patients in the same population (total in Canada: 59.5) [49]. Patients carrying *SOD1* mutations with bulbar onset, who usually present a later AAO, were less common than overall ALS patients in China according to our previous report [38] (8.1% vs 14%), which may partially explain why the mean AAO of *SOD1*-carrying patients was younger than that of the overall ALS patients in the same population.

Another striking finding of this study is the distinctive profile of disease progression and survival in the overall *SOD1*-mutant patients. The proportion of subpopulations was approximately 5:3:2 (slow: intermediate: fast). The overall median survival time of 89 months, much longer than that of 17.5 months reported in a recent *SOD*-related clinical trial [15], could be due to the rarity of the A4V mutation in the Chinese ALS population, which was characterized by a survival time of 1–2 years. The median survival of non-A4V *SOD1* patients in the United States was 6.8 years, comparable to that of our patients. Nevertheless, the discrepancy in the *SOD1*-related natural histories between Chinese and Caucasians should be recognized in the design and strategy of *SOD1*-targeting clinical trials.

The present study distinguished seven novel variants in the included ALS patients. These variants were evaluated as VUS or likely pathogenic according to the ACMG Standards and Guidelines [28] (Table 1). It is noteworthy that the nonsense variant of p.Glu133Ter was not classified as evidence of very strong pathogenicity because loss of function (LOF) is not believed to be the primary pathomechanism of *SOD1*-related ALS.

Since this cohort was based on a national referral site for ALS, caution should be applied when using these data to deduce the frequency of *SOD1* mutations in Chinese ALS patients. Additionally, the relatively younger mean AAO of *SOD1*-mutant patients and the longer median survival time compared with those of overall ALS patients in China (mean AAO: 43.92 years vs 49.8 years, median survival time: 89 months vs 71 months) made it more likely that these patients reported to the referral hospital. The calculated frequencies of 30% for fALS and 2% for sALS were likely an overestimation. Nevertheless, these numbers are the result of the greatest effort to date to explore the prevalence of *SOD1* mutations in the Chinese ALS population.

## Conclusions

In conclusion, the data shown herein demonstrate that p.H47R is the leading mutation in *SOD1*-mutant patients in China, and that female patients with *SOD1* mutations have notable advantages in disease progression and survival over male patients. Detailed analyses of patients’ natural histories revealed a discrepancy not only between Chinese and Caucasian ALS *SOD1*-mutant patients but also among different mutations. These findings may serve as a reference database and will be helpful for future *SOD1*-targeted clinical trials.

## Abbreviations

AAO: Age at onset
ACMG: American College of Medical Genetics
ALS: Amyotrophic lateral sclerosis
ALSFRS-R: ALS Functional Rating Scale Revised
ALSod: ALS Online database
C9orf72: Chromosome 9 open reading frame 72
db: Database
ExAC: Exome Aggregation Consortium
fALS: Familial amyotrophic lateral sclerosis
FUS: Fused in sarcoma
HGMD: Human Gene Mutation Database
HGVS: Human Genome Variation Society
IQR: Interquartile range
LOF: Loss of function
OPTN: Optineurin
PUTH: Peking University Third Hospital
sALS: Sporadic amyotrophic lateral sclerosis
SD: Standard deviation
SNP: Single nucleotide polymorphism
SOD1: Cu/Zn Superoxide dismutase 1
SQSTM1: Sequestosome 1
TARDBP: TAR DNA-binding protein-43
VUS: Variants of unknown significance
