# Correction to: Better survival in female *SOD1*-mutant patients with ALS: a study of *SOD1*-related natural history

**DOI:** 10.1186/s40035-019-0150-3

**Published:** 2019-03-19

**Authors:** Lu Tang, Yan Ma, Xiao-lu Liu, Lu Chen, Dong-sheng Fan

**Affiliations:** Department of Neurology, Peking University Third Hospital, 49 North Garden Road, Haidian District, Beijing, 100191 People’s Republic of China

**Correction to: Transl Neurodegener (2019) 8:2**

**https://doi.org/10.1186/s40035-018-0142-8**

In the original publication of this article [1], a numerical value in the sentence “The mean (SD) AAO was 43.92 years (9.24) for all subjects, with a significant difference between patients carrying mutations in exon 2 (*n* = 24, 46.83, 8.31) and exon 4 (*n* = 18, 37.75, 7.67) (*p* = 0.002).” is wrong. The true value in the parenthesis of exon 2 should be (*n* = 24, 46.63, 8.31).
